# SOX2-positive upper tract urothelial carcinoma: Clinicopathological characteristics and therapeutic implications

**DOI:** 10.3892/ol.2026.15629

**Published:** 2026-04-28

**Authors:** Hitomi Yasukawa, Yoshinori Ikehata, Naotaka Nishiyama, Hiroshi Kitamura

**Affiliations:** Department of Urology, Faculty of Medicine, University of Toyama, Toyama 930-0194, Japan

**Keywords:** SOX2, upper tract urothelial carcinoma, radical nephroureterectomy, perioperative chemotherapy, antibody-drug conjugate

## Abstract

Upper tract urothelial carcinoma (UTUC) is a rare subtype of urothelial malignancy associated with poor prognosis, particularly in advanced stages. Sex determining region-Y-related high mobility group box 2 (SOX2), a key transcription factor involved in the maintenance of cellular stemness, has been identified as a potential biomarker in multiple cancer types; however, its prognostic significance in UTUC remains unclear. The present study aimed to investigate the expression pattern of SOX2 in UTUC and examine its association with clinicopathological characteristics. Moreover, the present study aimed to evaluate the association of SOX2 with programmed cell death ligand 1 (PD-L1) and antibody-drug conjugate (ADC) targets, including Nectin-4 and trophoblast cell surface antigen 2, to explore potential therapeutic implications. A total of 87 patients with UTUC who underwent radical nephroureterectomy were retrospectively analyzed. SOX2 expression was assessed using immunohistochemistry with a 10% cut-off value. Notably, SOX2 expression was detected in 24% (21/87) of cases. No significant associations were observed between SOX2 expression and clinicopathological parameters, molecular subtypes, or the expression of PD-L1 and ADC targets, except for hydronephrosis grade. Patients with SOX2-positive tumors exhibited significantly worse overall survival (OS), cancer-specific survival (CSS) and recurrence-free survival (RFS; P=0.004, P=0.005 and P=0.011, respectively) during a median follow-up period of 39.3 months. The 5-year CSS rates were 69% in the SOX2-negative group and 43% in the SOX2-positive group. Multivariate analysis identified SOX2 expression as an independent prognostic factor for CSS (P=0.001). Among patients who did not receive perioperative chemotherapy, those with SOX2-positive tumors demonstrated significantly poorer OS, CSS and RFS, compared with SOX2-negative patients. These findings indicated that SOX2 expression represents an independent and robust prognostic biomarker in UTUC, identifying a biologically distinct high-risk subgroup with unfavorable clinical outcomes. The preserved expression of PD-L1 and ADC targets in this subgroup may suggest potential responsiveness to immune checkpoint inhibitors and ADC-based therapies, supporting the consideration of intensified perioperative or systemic treatment strategies in SOX2-positive patients.

## Introduction

Upper tract urothelial carcinoma (UTUC) is a relatively rare subtype of urothelial carcinoma, accounting for 5–10% of all urothelial malignancies. In Western countries, the estimated annual incidence is ~2 cases per 100,000 individuals (1). In comparison to urothelial carcinoma of the lower urinary tract, UTUC is frequently diagnosed at more advanced stages, largely due to anatomical factors that limit early detection and endoscopic surveillance. UTUC therefore represents a substantial clinical challenge, particularly in advanced disease. Patients with pathological stage T2 or T3 (pT2/pT3) tumors exhibit a 5-year overall survival (OS) rate of <50%, which declines to <10% in those with pT4 disease (2,3). Radical nephroureterectomy (RNU), the standard treatment for localized UTUC, remains essential for oncological control; however, this is associated with significant deterioration of renal function, further complicating subsequent management.

Advances in cancer biology have underscored the critical role of cancer stem cells (CSCs) in tumor initiation, progression, metastasis and therapeutic resistance (4). CSCs possess self-renewal capacity and multipotency, contributing to intratumoral heterogeneity and treatment failure through specific mechanisms, such as enhanced DNA repair, resistance to apoptosis and activation of drug efflux pathways. Sex determining region-Y-related high mobility group box 2 (SOX2), a transcription factor central to stemness maintenance, is widely recognized as a marker of CSCs in various malignancies (5). SOX2 regulates pluripotency-associated genes and self-renewal signaling pathways, and plays a pivotal role in embryonic development and stem cell biology. Together with other CSC markers, including aldehyde dehydrogenase 1 (ALDH1) (6), elevated SOX2 expression has been associated with aggressive tumor phenotypes and unfavorable clinical outcomes across multiple cancer types (7).

In UTUC, the results of preliminary studies suggested an association between CSC markers, including SOX2 and ALDH1, and adverse patient outcomes (8,9). However, these investigations were limited by small sample sizes and heterogeneous methodologies, resulting in inconsistent conclusions regarding the prognostic significance of individual CSC markers. The clinicopathological characteristics of CSC-positive UTUC, their molecular subtype distribution and the therapeutic implications of CSC-driven disease are yet to be fully elucidated. In addition, potential differences in response to systemic therapies, including chemotherapy and immunotherapy, between CSC-positive and CSC-negative UTUC have not been systematically examined. This knowledge gap limits risk stratification and the development of optimized treatment strategies for patients with biologically aggressive disease.

Given these limitations, the present study systematically evaluated SOX2 expression in UTUC and examined its association with clinicopathological features. The present study further assessed whether SOX2 expression functions as an independent prognostic biomarker beyond established clinicopathological parameters (10,11). In addition, the expression of programmed cell death ligand 1 (PD-L1) and antibody-drug conjugate (ADC) targets, including Nectin-4 and trophoblast cell surface antigen 2 (TROP-2), was analyzed to determine whether SOX2-positive UTUC represents a distinct therapeutic subgroup that may benefit from tailored systemic approaches. These findings may provide clinically relevant evidence to support improved risk stratification and personalized management in UTUC.

## Materials and methods

### Patients

The medical records of 87 consecutive patients with histologically confirmed UTUC who underwent RNU at Toyama University Hospital between January 2012 and December 2021 were retrospectively reviewed. Of these patients, 69 were male and 18 were female, with a median age of 74 years [interquartile range (IQR), 69–78; range, 51–87 years]. The present study was approved by the Institutional Review Board of the University of Toyama (approval no. R2019113). The requirement for informed consent was waived due to the retrospective design of the study and the use of anonymized data. Tumor staging was determined according to the 2017 Union for International Cancer Control (UICC) TNM classification system, and tumor grading was performed in accordance with the World Health Organization (WHO) 2016 grading criteria.

### Immunohistochemistry and scoring

**Figure 1.**
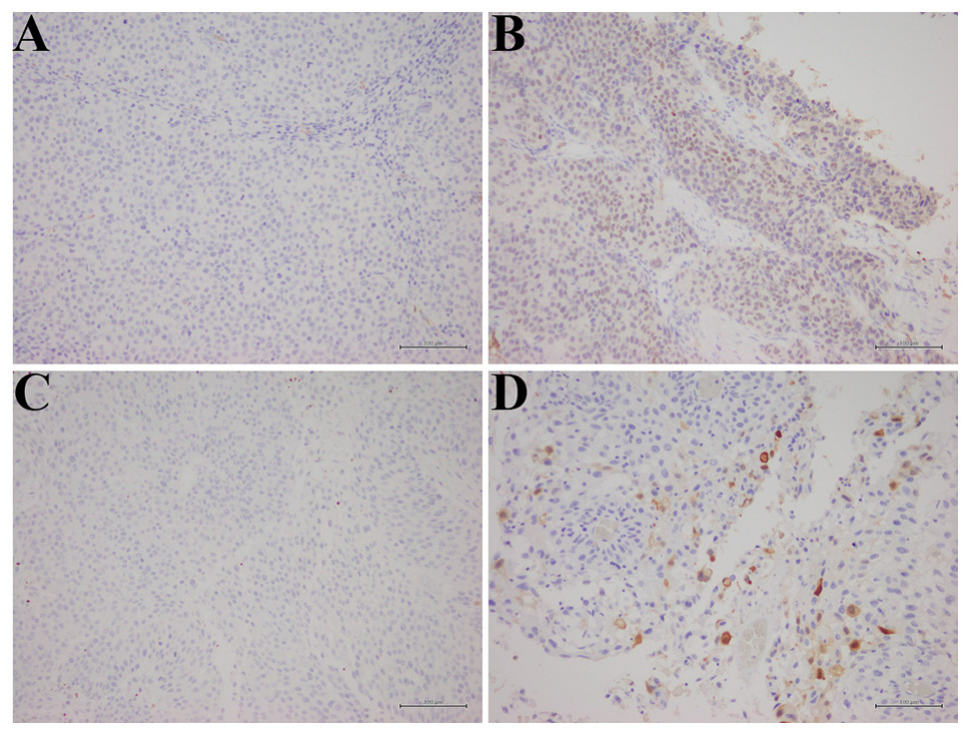
Representative immunohistochemical staining of SOX2 and ALDH1 in UTUC. (A) Negative SOX2 expression in tumor cells. (B) Positive SOX2 expression in tumor cells. (C) Negative ALDH1 expression in tumor cells. (D) Positive ALDH1 expression in tumor cells. Scale bar, 100 µm. UTUC, upper tract urothelial carcinoma; SOX2, sex determining region-Y-related high mobility group box 2; ALDH1, aldehyde dehydrogenase 1.

Formalin-fixed paraffin-embedded tumor sections (4 µm thick) underwent heat-induced epitope retrieval in Tris-EDTA buffer (pH, 9.0) in an autoclave prior to immunostaining with monoclonal antibodies against SOX2 (NBP2-29623, 1:200; Novus Biologicals, Ltd.; Bio-Techne) and ALDH1 (611194, 1:500; BD Transduction Laboratories; BD Biosciences). The chromogen was applied using Dako Envision FLEX+ (Agilent Technologies, Inc.), followed by counterstaining with hematoxylin, rinsing, dehydration and placing on a cover slip. Negative controls were prepared via substituting the primary antibody with dilution buffer. The criteria for determining positive and negative staining were based on previously published studies (12,13). For SOX2, a 10% cut-off defined positive and negative specimens (12). ALDH1 positivity was defined as staining in >1% of tumor cells (13). Representative immunohistochemical staining results for SOX2 and ALDH1 are displayed in Fig. 1.

**Figure 2.**
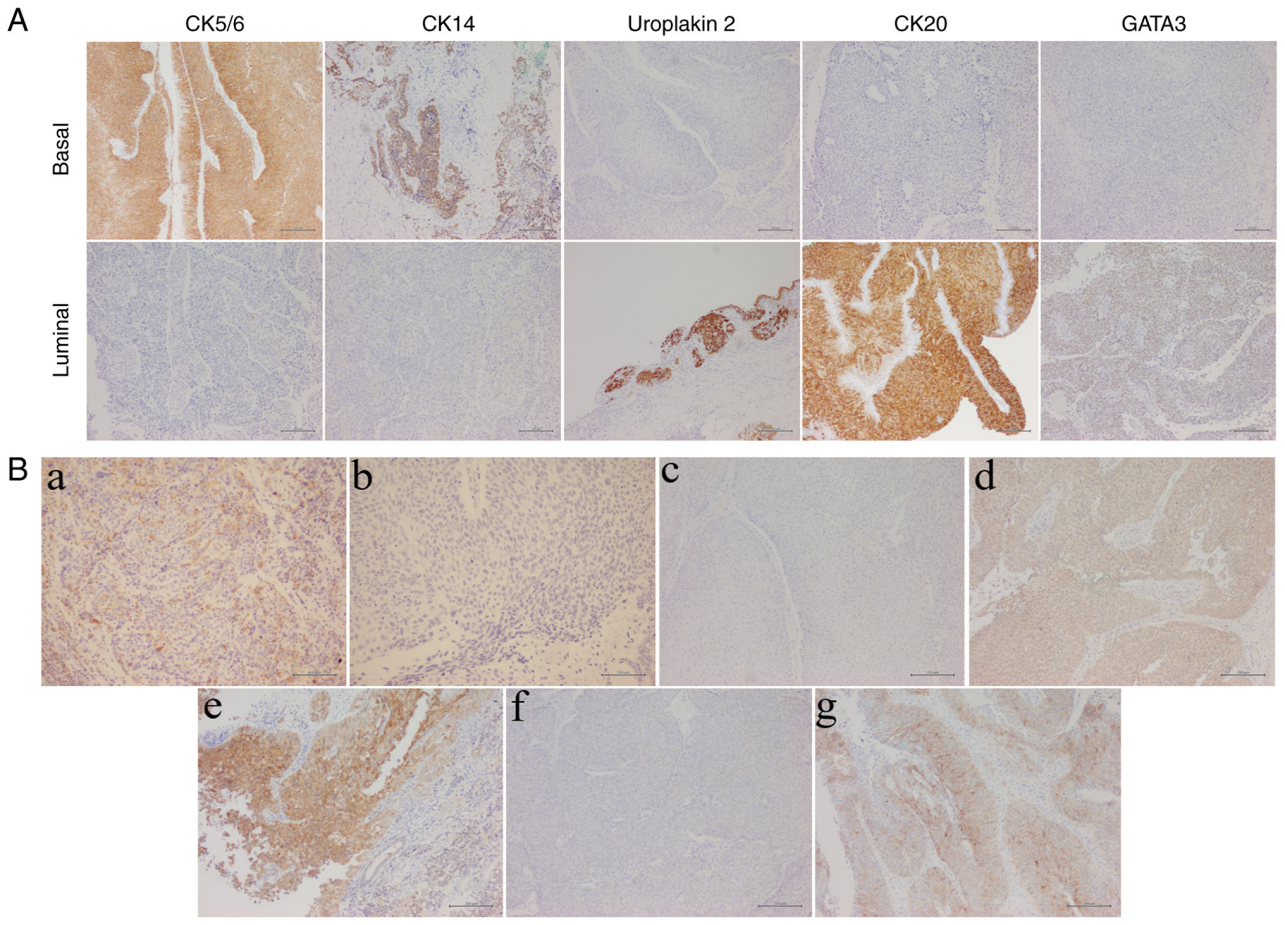
Immunohistochemical staining and molecular marker expression in UTUC. (A) Immunohistochemical staining patterns for molecular subtype markers (CK5/6, CK14, Uroplakin 2, CK20 and GATA3). Scale bar, 200 µm. (B) Immunohistochemical expression of PD-L1 and ADC targets (Nectin-4 and TROP-2) in UTUC tissue. (a) Negative PD-L1 expression. (b) Positive PD-L1 expression. Scale bar, 100 µm. (c) Negative Nectin-4 expression (intensity, 0). (d) Weak Nectin-4 expression (intensity, 1+). (e) Moderate Nectin-4 expression (intensity, 2+). (f) Negative TROP-2 expression. (g) Positive TROP-2 expression. Scale bar, 200 µm. UTUC, upper tract urothelial carcinoma; PD-L1, programmed cell death ligand 1; ADC, antibody-drug conjugate; TROP-2, trophoblast cell surface antigen 2; CK, cytokeratin.

Molecular subtypes were evaluated using antibodies against CK5/6 (418081, 1:1; Nichirei Biosciences, Inc.), CK20 (413491, 1:1; Nichirei Biosciences, Inc.), GATA3 (418201, 1:1; Nichirei Biosciences, Inc.), UPK2 (418121, 1:1; Nichirei Biosciences, Inc.) and CK14 (NCL-L-LL002, Leica, 1:50; Leica Biosystems; Fig. 2A) (14,15). Immunohistochemical scoring for all markers was based on the percentage of positive tumor cells. Molecular subtypes were hierarchically clustered using the average scores of CK20, GATA3 and UPK2 for the luminal type, and CK5/6 and CK14 for the basal type. Immunohistochemical analysis was also performed to assess PD-L1 (ab237726, 1:1,000; Abcam) and the ADC targets, Nectin-4 (ab192033, 1:4,000; Abcam) and TROP-2 (SC-376181, 1:1,000; Santa Cruz Biotechnology). A cut-off value of 1% was applied for PD-L1 expression in tumor cell membranes (Fig. 2B) (16). Nectin-4 expression was quantified using a H-score defined as the sum of staining intensity (0–3) multiplied by the percentage (0–100) of tumor cells at each intensity level. Specimens were classified as negative (H-score, 0–14) or positive (H-score, ≥15; Fig. 2B) (17). TROP-2 immunoreactivity localized to the cell membrane in ≥10% of tumor cells was considered positive (Fig. 2B) (18).

### Statistical analysis

OS, CSS and RFS were defined as the duration from RNU to death from any cause, cancer-specific death, or recurrence, respectively. Fisher's exact test was used to compare categorical variables between subgroups. The Mann-Whitney U test was applied to compare continuous variables. Survival curves were generated using the Kaplan-Meier method and compared using the log-rank test. The Cox proportional hazards model was used for univariate and multivariate analyses to identify mortality risk factors. Variables with P<0.05 in univariate analyses were entered into the multivariate model. All statistical tests were two-sided, and P<0.05 was considered to indicate a statistically significant difference. Statistical analyses were performed using SPSS (version, 27.0; IBM Corp.).

## Results

### Patient characteristics and positivity of cancer stem cell markers

Table I summarizes the characteristics of all 87 patients included in the present study. The median follow-up period was 39.3 months (interquartile range, 17.0–70.5 months). SOX2 and ALDH1 expression were positive in 24% (21/87) and 37% (32/87) of patients, respectively.

### Association between SOX2 expression and clinicopatholo-gical characteristics

Clinicopathological characteristics were compared between patients with SOX2-positive tumors and those with SOX2-negative tumors. No significant associations were identified between SOX2 expression and clinicopathological factors, with the exception of hydronephrosis. Moreover, no significant correlations were observed between SOX2 expression and molecular subtypes, PD-L1 expression or ADC targets, including Nectin-4 and TROP-2 (Table II).

### Survival outcomes and prognostic significance

**Figure 3.**
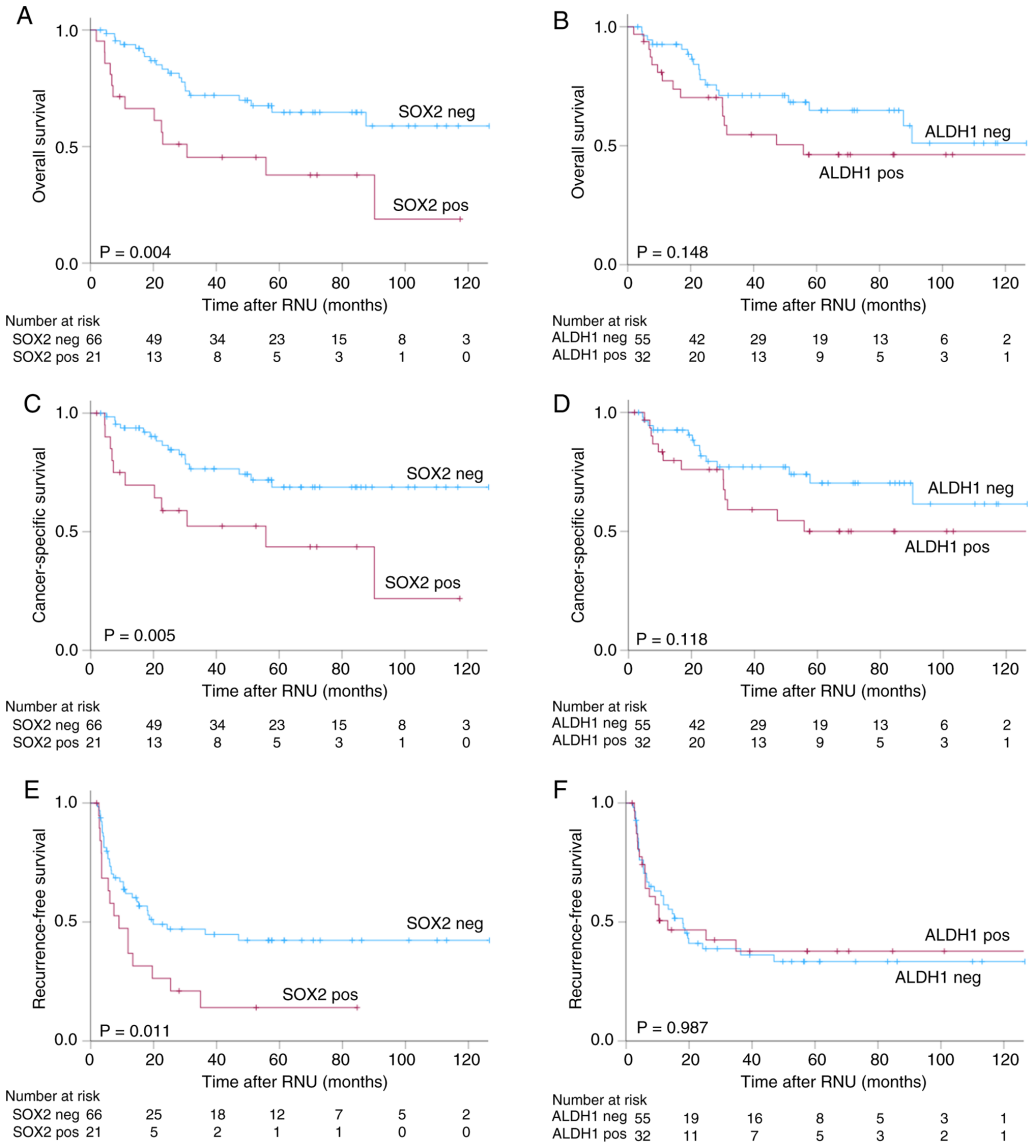
Kaplan-Meier curves for survival outcomes. Kaplan-Meier curves for overall survival according to (A) SOX2 expression status and (B) ALDH1 expression status; for cancer-specific survival according to (C) SOX2 expression status and (D) ALDH1 expression status; and for recurrence-free survival according to (E) SOX2 expression status and (F) ALDH1 expression status. SOX2, sex determining region-Y-related high mobility group box 2; ALDH1, aldehyde dehydrogenase 1.

In the overall cohort analysis, patients with SOX2-positive tumors exhibited significantly worse clinical outcomes than those with SOX2-negative tumors. SOX2 expression was associated with significantly shorter OS, CSS and RFS (P=0.004, P=0.005 and P=0.011, respectively; Fig. 3A, C and E). The 5-year OS, CSS and RFS rates in the SOX2-negative and SOX2-positive groups were 65 vs. 38%, 69 vs. 43% and 42 vs. 14%, respectively. By contrast, no significant differences were observed between the ALDH1-positive and ALDH1-negative groups with respect to OS, CSS or RFS (Fig. 3B, D and F).

### Univariate and multivariate analyses

The univariate analysis identified several factors associated with poor prognosis, including female sex, hydronephrosis grade, advanced pathological T stage, positive pathological N stage, lymphovascular invasion and SOX2 expression (Table III). Results of the multivariate analysis demonstrated that SOX2 expression acted as an independent prognostic indicator for CSS (P=0.001), demonstrating its prognostic value beyond conventional clinicopathological parameters (Table III).

### Treatment response analysis

**Figure 4.**
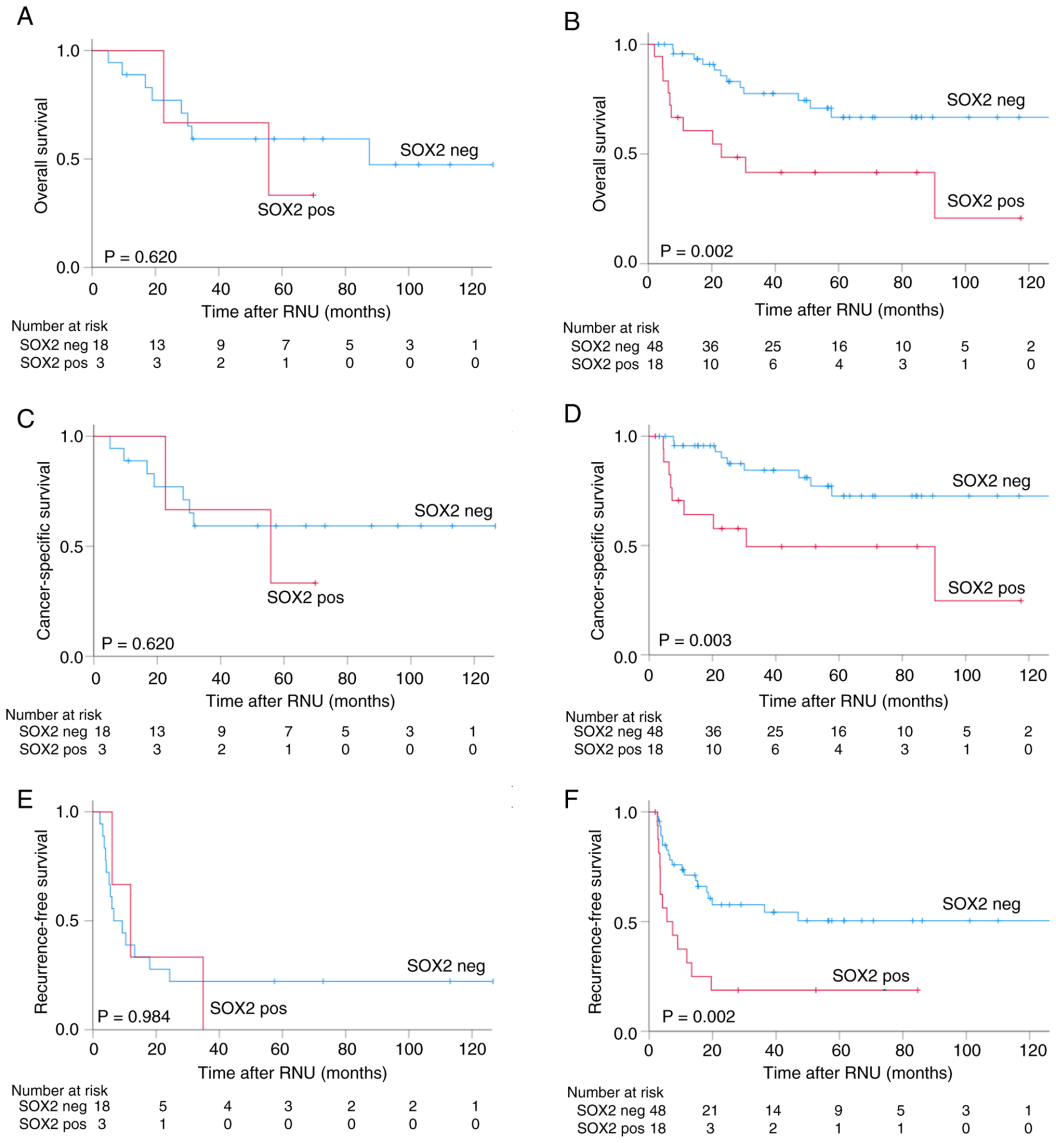
Kaplan-Meier curves illustrating (A) overall survival for patients who underwent NAC, AC or both according to SOX2 expression status. (B) Overall survival for patients who did not undergo NAC or AC. (C) Cancer-specific survival for patients with NAC or AC. (D) Cancer-specific survival for patients without NAC or AC. (E) Recurrence-free survival for patients with NAC or AC. (F) Recurrence-free survival for patients without NAC or AC. NAC, neoadjuvant chemotherapy; AC, adjuvant chemotherapy; SOX2, sex determining region-Y-related high mobility group box 2.

Impact of SOX2 expression on treatment response was analyzed via comparing the outcomes of patients who received or did not receive platinum-based chemotherapy (neoadjuvant, adjuvant or both; Fig. 4). Among patients who did not receive neoadjuvant or adjuvant chemotherapy, those with SOX2-positive tumors consistently showed poorer outcomes than those with SOX2-negative tumors. The 5-year CSS rate was 73% in patients with SOX2-negative tumors, vs. 49% in those with SOX2-positive tumors (Fig. 4D). The 5-year RFS rates were 50 and 19%, respectively (Fig. 4F).

## Discussion

The present study provides a comprehensive analysis demonstrating that SOX2 expression is a strong and independent prognostic biomarker in patients with UTUC undergoing radical nephroureterectomy. A clinically significant difference in 5-year CSS was observed (43% for SOX2-positive vs. 69% for SOX2-negative), underscoring the importance of identifying this highly aggressive patient subset. SOX2 expression remained an independent prognostic factor in multivariate analyses, irrespective of conventional clinicopathological variables, such as tumor stage and grade. This independence suggests that SOX2 may define a biologically distinct disease entity characterized by high stemness and intrinsic malignancy, independent of morphological tumor characteristics.

In the present cohort, the prevalence of SOX2 expression (24%) was consistent with previous reports in urothelial carcinomas, where SOX2 positivity ranged from 15 to 30% (8,9). The 26% absolute difference in CSS may represent a clinically meaningful prognostic distinction with implications for treatment planning and patient counseling. Multivariate analyses (P=0.001) confirmed that SOX2 may provide prognostic data beyond established parameters, such as tumor stage, grade and lymphovascular invasion (19). Notably, SOX2 expression did not correlate with traditional prognostic variables, further supporting its role in identifying a biologically distinct, intrinsically aggressive subset of UTUC. The function of SOX2 as a master regulator of stemness and pluripotency provides biological plausibility for its association with poor prognosis (5).

The unfavorable outcomes observed in patients with SOX2-positive UTUC emphasized the requirement for novel therapeutic strategies targeting this high-risk population. CSCs, characterized by self-renewal and multilineage differentiation capacities, frequently exhibit resistance to conventional anticancer agents and radiotherapy (20). SOX2 positivity may reflect enhanced tumor-initiating capacity, increased resistance to systemic therapies and a greater metastatic potential (21).

Recent advances in molecular classification have stratified muscle-invasive urothelial carcinoma (MIUC) into distinct molecular subtypes with differing prognoses (22). Emerging evidence indicated that SOX2 regulates key genes within the basal/squamous (Ba/Sq) subtype of MIUC and modulates chemotherapeutic response (23). Results of a previous study demonstrated that SOX2 depletion reduced Ba/Sq markers, increased luminal markers and enhanced cisplatin sensitivity in MIUC (23). Although UTUC and bladder urothelial carcinoma differ in epidemiological and clinicopathological characteristics (24), therapeutic strategies targeting SOX2 may also be applicable to UTUC through modulating molecular subtype features and improving chemotherapy responsiveness. These findings raise the possibility that neoadjuvant chemotherapy (NAC), historically lacking clearly established efficacy in UTUC (25), may confer benefit when combined with SOX2-targeted strategies. If SOX2 inhibition enhances cisplatin efficacy, as demonstrated in bladder cancer models, combination approaches may improve outcomes in patients with SOX2-positive UTUC.

To the best of our knowledge, the present study is the first to evaluate the association between SOX2 expression and therapeutic biomarkers, including PD-L1, and ADC targets; namely, Nectin-4 and TROP-2, in a UTUC cohort undergoing RNU. Comparable expression levels of PD-L1, Nectin-4 and TROP-2 between SOX2-positive and SOX2-negative tumors suggest that immune checkpoint inhibitors (ICIs) and ADC therapies, including enfortumab vedotin and sacituzumab govitecan, may remain effective treatment options in this high-risk subgroup.

Notably, the present study exhibits limitations. The retrospective, single-institution design and relatively small sample size may limit generalizability, and validation in larger, multi-center cohorts is required. Although the prognostic significance of SOX2 was demonstrated, the molecular mechanisms underlying the adverse outcomes in SOX2-positive patients remain to be fully elucidated. Treatment response analysis was limited by chemotherapy heterogeneity and retrospective treatment selection. Furthermore, we were unable to assess longitudinal changes in SOX2 expression following NAC or at the time of recurrence. Although four patients received NAC, pre-treatment biopsy specimens were available for only one case [the remaining three were diagnosed via urinary cytology (n=2) or imaging (n=1)]. Consequently, we were unable to evaluate the impact of NAC on SOX2 expression in the current cohort. This remains an important subject for future investigations. In addition, the association between SOX2 expression and specific molecular subtypes of UTUC, as well as the feasibility of SOX2-targeted therapies, should be further explored in prospective studies. While the present study was limited to an immunohistochemistry-based classification for molecular subtype assessment, previously proposed molecular classifications based on whole-exome sequencing require further investigation to clarify their association with SOX2 expression (26,27).

Although the present study included a single-institution retrospective analysis, a limitation common in studies of rare malignancies such as UTUC, the statistical robustness of SOX2 as an independent prognostic factor supports its clinical relevance. Moreover, the present study provided the first evidence evaluating the association between SOX2 expression and PD-L1 and ADC targets. The comparable expression of these markers in SOX2-positive and SOX2-negative tumors suggested that ICIs and ADC therapies, such as enfortumab vedotin or sacituzumab govitecan, may represent viable therapeutic options for this high-risk subgroup, offering an immediate and clinically meaningful treatment opportunity.

## Figures and Tables

**Table I.** Patient characteristics (N=87).

Characteristic	n (%) or median (IQR)
Median age, years	74 (69–78)
Sex	
Male	69 (79)
Female	18 (21)
ECOG PS	
0/1	73 (84)
2-4	14 (16)
Renal function, eGFR (ml/min/1.73 m^2^)	
≥60	34 (39)
45-59	22 (25)
<45	31 (36)
Primary site	
Renal pelvis	38 (44)
Ureter	44 (51)
Both	5 (6)
Side	
Right	49 (56)
Left	36 (41)
Bilateral	2 (2)
Hydronephrosis grade	
0/1	33 (38)
2-4	54 (62)
Pathological T stage	
pTis/pTa/pT1	34 (39)
pT2	10 (12)
pT3	41 (47)
pT4	2 (2)
Pathological N stage	
pN-	23 (26)
pN+	8 (9)
pNx	56 (64)
Tumor grade	
Low grade	16 (18)
High grade	66 (76)
Unknown	5 (6)
Lymphovascular invasion	
Negative	46 (53)
Positive	29 (33)
Unknown	12 (14)
Tumor histology	
Pure UC	82 (94)
UC with variant histology	5 (6)
Squamous differentiation	3 (3)
Glandular differentiation	1 (1)
Sarcomatoid variant	1 (1)
History of bladder cancer	
No	74 (85)
Yes	13 (15)
Perioperative chemotherapy	
Neoadjuvant chemotherapy	
No	83 (95)
Yes	4 (5)
GEM + CDDP	3 (3)
GEM + PTX	1 (1)
Adjuvant chemotherapy	
No	69 (79)
Yes	18 (21)
GEM + CDDP	16 (18)
GEM + CBDCA	2 (2)

IQR, interquartile range; ECOG PS, Eastern Cooperative Oncology Group performance status; eGFR, estimated glomerular filtration rate; UC, urothelial carcinoma; GEM, gemcitabine; CDDP, cisplatin; PTX, paclitaxel; CBDCA, carboplatin.

**Table II.** Associations between SOX2 expression and clinicopathological characteristics.

Characteristic	SOX2 positive (n=21)	SOX2 negative (n=66)	P-value
Median age, years	74 (65–77)	75 (70–78)	0.188
Sex			
Male	18 (86)	51 (77)	0.543
Female	3 (14)	15 (23)	
ECOG PS			
0/1	15 (71)	58 (88)	0.078
2-4	6 (29)	8 (12)	
eGFR (ml/min/1.73 m^2^)			
≥45	11 (52)	45 (68)	0.202
<45	10 (48)	21 (32)	
Primary site			
Renal pelvis	7 (33)	31 (47)	0.490
Ureter	13 (62)	31 (47)	
Both	1 (5)	4 (6)	
Bilateral disease			
Unilateral	21 (100)	64 (97)	1.000
Bilateral	0 (0)	2 (3)	
Hydronephrosis grade			
0/1	3 (14)	30 (45)	0.011
2-4	18 (86)	36 (55)	
pT stage			
pTis/pTa/pT1	8 (38)	26 (39)	0.574
pT2	1 (5)	9 (14)	
pT3	11 (52)	30 (45)	
pT4	1 (5)	1 (2)	
pN stage			
pN-	7 (33)	16 (24)	0.388
pN+	3 (14)	5 (8)	
pNx	11 (52)	45 (68)	
Tumor grade			
Low grade	4 (19)	12 (18)	0.434
High grade	13 (62)	53 (80)	
Unknown	4 (19)	1 (2)	
Lymphovascular invasion			
Negative	11 (52)	35 (53)	0.982
Positive	7 (33)	22 (33)	
Unknown	3 (14)	9 (14)	
Tumor histology			
Pure UC	20 (95)	62 (94)	0.824
UC with variant histology	1 (5)	4 (6)	
Squamous differentiation	1 (5)	2 (3)	
Glandular differentiation	0	1 (2)	
Sarcomatoid variant	0	1 (2)	
Molecular subtype			
Luminal	14 (67)	52 (79)	0.258
Basal	7 (33)	14 (21)	
PD-L1			
Positive	6 (29)	22 (33)	0.792
Negative	15 (71)	44 (67)	
ADC targets			
Nectin-4			
Positive	17 (81)	56 (85)	0.736
Negative	4 (19)	10 (15)	
TROP-2			
Positive	17 (81)	52 (79)	1.000
Negative	4 (19)	14 (21)	

Data are presented as n (%) or median (IQR). SOX2, sex determining region-Y-related high mobility group box 2; IQR, interquartile range; ECOG PS, Eastern Cooperative Oncology Group performance status; eGFR, estimated glomerular filtration rate; UC, urothelial carcinoma; PD-L1, programmed cell death ligand 1; ADC, antibody-drug conjugates; TROP-2, trophoblast cell surface antigen 2.

**Table III.** Prognostic factors for cancer-specific survival in univariate and multivariate analyses.

	Univariate analysis		Multivariate analysis	
		
Factor	HR (95% CI)	P-value	HR (95% CI)	P-value
Age, ≥75 vs. <75	0.855 (0.399–1.829)	0.855	N/A	N/A
Sex, male vs. female	0.305 (0.141–0.661)	0.003^a^	0.286 (0.093–0.881)	0.029^a^
ECOG PS, 2–4 vs. 0/1	0.709 (0.459–1.095)	0.143	N/A	N/A
Reduced renal function, eGFR <45 vs. ≥45	0.733 (0.501–1.071)	0.121	N/A	N/A
Hydronephrosis grade, 2–4 vs. 0/1	2.868 (1.156–7.119)	0.014^a^	1.117 (0.648–1.924)	0.691
pT stage, ≥ pT2 vs. ≤ pT1	3.686 (1.390–9.772)	0.009^a^	1.777 (0.459–7.019)	0.412
pN stage, pN+ vs. pN-	5.632 (2.215–14.316)	<0.001^a^	4.591 (1.510–13.957)	0.007^a^
Tumor grade, high vs. low	1.590 (0.546–4.624)	0.370	N/A	N/A
LVI, positive vs. negative	4.509 (1.921–10.580)	<0.001^a^	2.173 (0.754–6.265)	0.151
History of bladder cancer, yes vs. no	0.724 (0.218–2.407)	0.599	N/A	N/A
SOX2, positive vs. negative	2.850 (1.321–6.148)	0.008^a^	4.820 (1.842–12.614)	0.001^a^
ALDH1, positive vs. negative	1.812 (0.851–3.859)	0.123	N/A	N/A
Molecular subtype, basal vs. luminal	1.663 (0.727–3.801)	0.228	N/A	N/A
PD-L1, positive vs. negative	1.841 (0.853–3.974)	0.120	N/A	N/A

a P<0.05. HR, hazard ratio; CI, confidence interval; ECOG PS, Eastern Cooperative Oncology Group performance status; eGFR, estimated glomerular filtration rate; LVI, lymphovascular invasion; SOX2, sex determining region-Y-related high mobility group box 2; ALDH1, aldehyde dehydrogenase 1; PD-L1, programmed cell death ligand 1.

## Data Availability

The data generated in the present study may be requested from the corresponding author.

## Abbreviations

UTUC: upper tract urothelial carcinoma
RNU: radical nephroureterectomy
CSC: cancer stem cell
SOX2: sex determining region-Y-related high mobility group box 2
ALDH1: aldehyde dehydrogenase 1
PD-L1: programmed cell death ligand 1
ADC: antibody-drug conjugate
TROP-2: trophoblast cell surface antigen 2
CK: cytokeratin
UPK2: uroplakin 2
H-score: histochemical score
OS: overall survival
CSS: cancer-specific survival
RFS: recurrence-free survival
MIUC: muscle-invasive urothelial carcinoma
Ba/Sq: basal/squamous
NAC: neoadjuvant chemotherapy
ICIs: immune checkpoint inhibitors
